# Age-gender reference values of native myocardial T1 at 1.5T and 3T: comparison of MOLLI and shMOLLI

**DOI:** 10.1186/1532-429X-15-S1-P91

**Published:** 2013-01-30

**Authors:** Darius Dabir, Toby Rogers, Tobias Voigt, Tobias Schaeffter, Eike Nagel, Valentina O Puntmann

**Affiliations:** 1 Cardiovascular Imaging, King's College London, London, UK; 2 Philips Innovative Technologies, Clinical Research, London, UK; 3 Medical Physics and Bioengineering, King's College London, London, UK

## Background

Native T1 mapping is promising non-invasive method for quantitative assessment of diffuse myocardial processes. The aim of the current study was to interrogate normal values for T1 measurement within native myocardium for age and gender using most commonly used sequences and field strengths.

## Methods

Ninety-nine subjects with no history of cardiovascular or systemic disease or regular medication underwent native T1 imaging with modified (MOLLI; 3,3,5) and shortened modified look-locker inversion recovery sequence (shMOLLI; 1,1,5) at either 1.5T or 3T. The images were analyzed using an Osirix plug-in with manual motion correction of the ROI placed conservatively within the septal myocardium. For comparison, lateral myocardium and blood values were also derived.

## Results

There was similar representation for age and gender across both field strengths (Table 1). Mean value for T1 native myocardial by MOLLI was higher than shMOLLI for septum and lateral ROI for both field strengths, p<0.01 for all). There was no significant difference for gender nor age-groups in T1 relaxation time for either MOLLI or shMOLLI imaging at either field strength.

**Table 1:** 

Population	1.5T n=47	3T n=52
Age (years)	39±13	39±14

Male (n,%)	26 (55)	28 (53)

MOLLI		

Septum (msec)	943±45	1055±39
Lateral (msec)	917±55	1025±55
Blood (msec)	1594±111	1759±181

shMOLLI		

Septum (msec)	833±55	894±74
Lateral (msec)	800±61	854±84
Blood (msec)	1554±210	1662±120

Age groups		

MOLLI (septal values)		

<30 years	944±50	1060±35
30-45 years	943±42	1046±44
>45 years	946±32	1038±32

shMOLLI (septal values)		

<30 years	837±57	869±65
30-45 years	846±140	851±120
>45 years	840 ±47	912±82

Gender (septal values)		

MOLLI		

Male (msec)	945±44	1057±37
Female (msec)	942±50	1050±37

shMOLLI		

Male (msec)	839±45	885±91
Female (msec)	846±57	878±84

## Conclusions

We report age-gender normal values for native myocardial T1 mapping by the two most commonly used sequences and field strenghts. We show that there are neither gender differences in a cohort as a whole nor within separate age groups.

## Funding

NIHR

**Figure 1:**
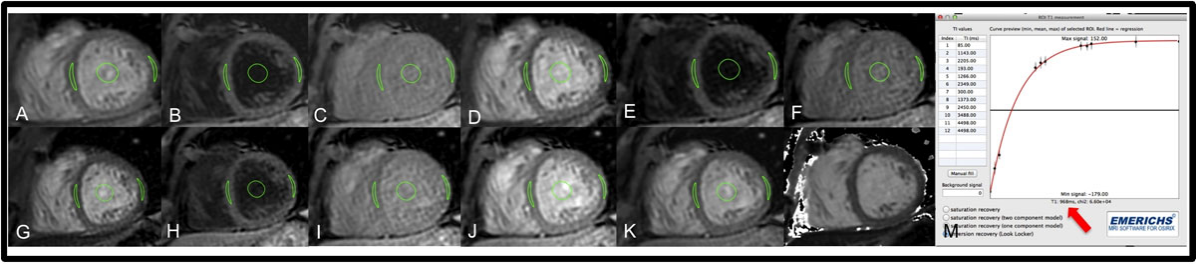
T1 source images (A-K) as well as the T1 map (L) acquired in mid-ventricular short-axis slice. ROI placements within septum, lateral wall and blood pool (green marks). Image M shows the analyzed septal T1 relaxation time (red arrow).

